# Feasibility of the Four ‘A’s Test (4AT) Delirium Screening Tool in Older Adults Presenting to the Emergency Department: A Rapid Systematic Review

**DOI:** 10.7759/cureus.114359

**Published:** 2026-08-11

**Authors:** William De Vivo, Chioma Onuha

**Affiliations:** 1 Geriatrics, Salford Royal Hospital, Salford, GBR; 2 Emergency Medicine, Salford Royal Hospital, Salford, GBR

**Keywords:** 4at, assessment tool feasibility, delirium screening, elderly people, emergency department(ed)

## Abstract

Delirium is a common, serious, and frequently underdiagnosed condition in older adults presenting to the emergency department (ED). The Four ‘A’s Test (4AT) is a brief, validated delirium screening tool requiring no specialist training or equipment, making it potentially well-suited to the fast-paced ED environment. However, evidence on its feasibility in this setting remains limited and has not been systematically synthesised. This rapid systematic review aimed to evaluate the feasibility of the 4AT, including completion time, completion rates, staff acceptability, and implementation barriers and facilitators, in EDs for adults aged 65 years and older.

A rapid systematic review was conducted in accordance with PRISMA 2020 guidelines and prospectively registered on PROSPERO (CRD420261370525). PubMed, MEDLINE via Ovid, and EMBASE via Ovid were searched from January 2011 to April 2026. Two reviewers independently screened titles, abstracts, and full texts, with conflicts resolved by discussion. Primary studies reporting feasibility outcomes for the 4AT in an ED setting in adults aged ≥65 were included. Data were extracted using a standardised form and methodological quality was assessed using the Joanna Briggs Institute (JBI) critical appraisal checklist appropriate to each study design. Findings were synthesised narratively.

A total of 195 records were identified across PubMed, MEDLINE, and EMBASE. Following removal of 98 duplicates, 97 unique records were screened at title and abstract stage, of which 23 were retrieved for full-text review. Six studies met the inclusion criteria and were included in the final synthesis (UK n = 3, Spain n = 1, Norway n = 1, Netherlands n = 1). The available evidence suggests that the 4AT is feasible across emergency care settings, with short completion times, high acceptability among healthcare professionals, and minimal training requirements. Implementation success was influenced primarily by organisational factors, with education, electronic integration, and workflow support facilitating uptake, while time pressures and competing clinical demands remained key barriers.

The available evidence suggests that the 4AT is a feasible delirium screening tool for older adults presenting to EDs, demonstrating rapid administration, minimal training requirements, and generally positive staff acceptability. However, successful implementation appears to depend on organisational factors, including staff education, workflow integration, and clear allocation of screening responsibilities. These findings support consideration of the 4AT within ED delirium screening pathways, while highlighting the need for further research on implementation strategies and long-term clinical outcomes.

## Introduction and background

Background

Delirium is an acute neuropsychiatric syndrome characterised by disturbances in attention, awareness, and cognition that develop over a short period of time and tend to fluctuate throughout the day. It is common in older adults presenting to the emergency department (ED), with reported prevalence rates ranging from 7% to 20% in this population, rising to over 40% in those with pre-existing cognitive impairment [1]. Despite its frequency, delirium is estimated to be missed in up to 70% of cases in the ED, with studies consistently demonstrating that hypoactive delirium in particular is poorly recognised by clinical staff without the use of a structured screening tool [2].

The consequences of missed or delayed delirium diagnosis are significant. Delirium in older adults is associated with prolonged hospital length of stay, increased rates of institutionalisation, accelerated cognitive decline, and higher short and long-term mortality [3]. Early identification therefore has direct implications for patient safety, management decisions, and downstream outcomes. Despite this, routine delirium screening in the ED remains inconsistent across healthcare systems, including within the UK [4].

The Four ‘A’s Test (4AT), developed by MacLullich and colleagues, has emerged as a particularly promising delirium screening tool for ED use, with its validation first published by Bellelli et al. in 2014 [5]. It comprises four items: alertness, abbreviated mental test score, attention, and acute change or fluctuation, and can typically be completed in under two minutes without specialist training or equipment [5]. Evidence on its diagnostic accuracy is growing, with studies reporting sensitivity of 76-89% and specificity of 72-95% against reference standard diagnosis [6].

Rationale

Diagnostic accuracy alone does not determine whether a screening tool can be successfully implemented in a real-world ED environment. Feasibility, encompassing completion time, completion rates, staff acceptability, training requirements, and implementation barriers and facilitators, is a distinct and critical consideration, particularly in the ED context. EDs are characterised by high patient throughput, time pressure, frequent interruptions, and a workforce not primarily trained in delirium assessment. A tool may perform well under controlled research conditions yet prove impractical in routine clinical use. Understanding feasibility is therefore a prerequisite for effective, sustainable implementation of 4AT screening at scale.

To date, no systematic review has specifically synthesised the feasibility evidence for the 4AT in the ED. Existing reviews have focused predominantly on diagnostic accuracy across inpatient settings, with ED-specific feasibility data either absent or reported only incidentally [7]. This represents a significant gap in the evidence base and a barrier to implementation guidance for ED clinicians and service designers seeking to introduce routine delirium screening for older adults.

Objectives

This rapid systematic review has two objectives. The primary objective is to synthesise the available evidence on the feasibility of the 4AT delirium screening tool in EDs for adults aged 65 years and older, with specific focus on completion time, completion rates, staff acceptability, training requirements, and barriers and facilitators to 4AT implementation in the ED. The secondary objective is to extract diagnostic accuracy data where reported alongside feasibility outcomes, in order to contextualise feasibility findings within the broader evidence base.

## Review

Methods 

Protocol and Registration

This rapid systematic review was prospectively registered on PROSPERO (registration number CRD420261370525) and was conducted and reported in accordance with the PRISMA 2020 guidelines.

Eligibility Criteria

This review was structured using the PICO framework. The population of interest was adults aged 65 years and older presenting to any ED. The index tool (intervention) was the 4AT assessment instrument in its standard form. No formal comparator was specified, though studies reporting diagnostic accuracy data alongside feasibility outcomes were eligible, and these data were extracted as a secondary outcome. The primary outcomes of interest were feasibility measures including completion time, completion rates, staff acceptability, training requirements, and barriers and facilitators to implementation. Secondary outcomes were sensitivity and specificity of the 4AT for delirium testing. A summary of the PICO framework underpinning the review question is provided in Appendix 1.

Table 1 illustrates the inclusion and exclusion criteria used for this systematic review.

**Table 1:** Table demonstrating the inclusion and exclusion criteria used when screening articles during this review

Inclusion Criteria	Exclusion Criteria
4AT administered in an ED setting	4AT used outside the ED (ward, ICU, community, care home)
Primary studies (any design)	Reviews, editorials, letters, protocols, conference abstracts only
Participants aged ≥65 years	Age <65 (unless ≥65 subgroup extractable)
Reports ≥1 feasibility outcome (completion time, completion rate, staff acceptability, training requirements)	Diagnostic accuracy data only - no feasibility data reported
English language, 2011 to present	Non-English language without translation available

Information Sources

A systematic search of three electronic databases was conducted: PubMed, MEDLINE via Ovid, and EMBASE via Ovid. The search strategy was developed iteratively and combined terms relating to three concept domains: the 4AT tool, the ED setting, and older adults. For MEDLINE and EMBASE via Ovid, MeSH and Emtree terms, respectively, were exploded where appropriate and combined with free-text synonyms using field codes. For PubMed, an equivalent single-line Boolean search string was used, combining the same concept domains with publication type and date filters applied. The full search strategies for all three databases are provided in Appendix 2. No grey literature search was conducted given the rapid nature of this review; this is acknowledged as a limitation. Reference lists of all included studies were hand-searched to identify any additional eligible studies.

Article Screening

All records identified through searches of PubMed, MEDLINE, and EMBASE were exported and imported into Rayyan (rayyan.ai) (Rayyan Systems Inc., Cambridge, MA) for deduplication and screening. A total of 195 records were identified prior to deduplication; 98 duplicate records were removed, leaving 97 unique records for title and abstract screening. Automated deduplication was performed within Rayyan, followed by manual verification of flagged duplicates. Title and abstract screening was conducted independently by two reviewers (WDV and CO) using blind mode in Rayyan, whereby decisions were concealed from each reviewer until the reveal stage. Conflicts were resolved by discussion and consensus. Twenty-three records were retrieved for full-text review, which was subsequently performed independently by both reviewers, with disagreements again resolved by discussion. Reasons for exclusion at the full-text stage were recorded and are reported in the PRISMA flow diagram. Six studies were identified as meeting the inclusion criteria and were carried forward to data extraction.

Data Extraction

Data were extracted from all six included studies using a standardised extraction form developed a priori by the review team. Two reviewers independently extracted data from all included studies. Discrepancies were resolved by discussion. The following data were extracted: study identification details (author, year, country); study design; ED setting; sample size and participant demographics; details of 4AT administration including who administered the tool, training received, and timing of assessment within the ED pathway; primary feasibility outcomes (completion time, completion rate, staff acceptability, training requirements); barriers and facilitators to implementation; and where reported, secondary diagnostic accuracy outcomes (sensitivity, specificity, reference standard used).

Quality Appraisal

Methodological quality was assessed using the appropriate Joanna Briggs Institute (JBI) critical appraisal checklist for each study design. Relevant domains included the clarity of inclusion and exclusion criteria, representativeness of the sample, description of 4AT administration procedures, validity and reliability of outcome measurement, management of confounding factors, handling of missing data, and appropriateness of statistical analysis.

Each checklist item was assessed as "Yes", "No", "Unclear", or "Not Applicable" in accordance with JBI guidance. Consistent with JBI methodology, no numerical scoring system was applied. Instead, an overall judgement of low, moderate, or high risk of bias was made based on consideration of all checklist domains and the likely impact of any methodological limitations on the validity of the study findings.

Studies were judged as low risk of bias when most appraisal criteria were met, and any limitations were unlikely to meaningfully affect the findings. Studies were judged as moderate risk of bias when some criteria were unmet or unclear and may have influenced study conclusions. High-risk studies were those with multiple important methodological limitations that raised substantial concerns regarding the validity of the findings. Quality appraisal findings were used to inform interpretation of the evidence but were not used as a basis for study exclusion.

Data Synthesis

Given the anticipated heterogeneity in study design and variability in how feasibility outcomes were measured and reported across included studies, a meta-analysis was not planned or conducted. Findings were synthesised narratively, organised thematically by primary feasibility outcome. Where multiple studies reported quantitative feasibility data for the same outcome, these are presented descriptively and compared. Risk of bias ratings are reported alongside study findings to contextualise the strength of the evidence.

Results

Study Selection

A total of 195 records were identified through searches of three electronic databases: PubMed (n = 25), MEDLINE via Ovid (n = 30), and EMBASE via Ovid (n = 140). Following automated and manual deduplication within Rayyan, 98 duplicate records were removed, leaving 97 unique records for title and abstract screening. Two reviewers independently screened all 97 records in blind mode, with conflicts resolved by discussion and consensus. Following this stage, 74 records were excluded as not meeting the inclusion criteria based on title and abstract content, and 23 records were retrieved for full-text review.

**Figure 1:**
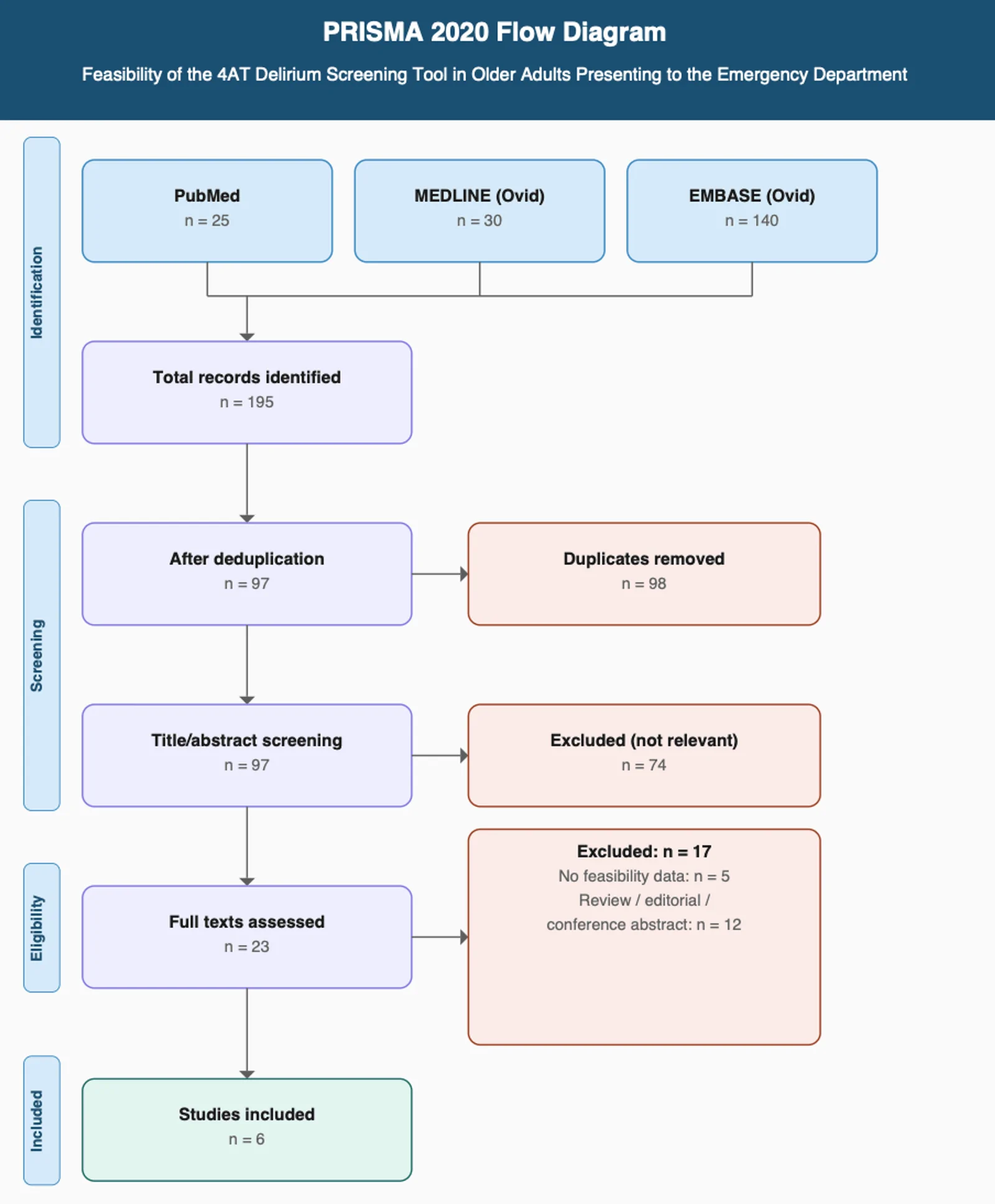
PRISMA diagram demonstrating articles that were identified and eventually included in this systematic review

Full-text assessment was conducted independently by both reviewers. Of the 23 full texts assessed for eligibility, 17 were excluded: five were excluded initially due to absence of feasibility outcome data, reporting diagnostic accuracy of the 4AT only, with the remaining 12 being excluded as they were reviews, editorials, or conference abstracts. Further details of this are available in Appendix 3. Six studies met all inclusion criteria and were included in the final synthesis. The study selection process is summarised in the PRISMA 2020 flow diagram (Figure 1).

Study Characteristics

The characteristics of the six included studies are presented in Table 2. Publication dates ranged from 2019 to 2025, with studies conducted in four countries: the UK (n = 3), Spain (n = 1), Norway (n = 1), and the Netherlands (n = 1). Sample sizes varied substantially, ranging from 49 to 785 participants. The six included studies comprised Soler-Sanchis et al. [8], Pouw et al. [9], Myrstad et al. [10], Durant and Karia [11], MacLullich et al. [12], and Vardy et al. [13]. The combined sample size across the six studies was 1,583 participants; however, this figure should be interpreted with caution, as Pouw et al. recruited from both ED and geriatric ward settings and did not report an ED-only subgroup size separately [9], and Vardy et al. did not report a sample size at all [13]. As a result, the true number of participants assessed specifically within EDs cannot be precisely determined from the available data.

**Table 2:** Table showing characteristics of studies included in this systematic review

Study	Country	Design	Setting	Sample Size	Mean Age (Years)	Risk of Bias
Soler-Sanchis et al., 2024	Spain	Diagnostic accuracy	Emergency Department	370	81.8	Low
Pouw et al., 2023	Netherlands	Prospective cohort	Emergency Department and Geriatric Wards	49	75	Moderate
Myrstad et al., 2022	Norway	Prospective cohort	Emergency Department	229	81	Low
Durant and Karia, 2025	United Kingdom	Quality improvement	Emergency Department	150	NR	High
MacLullich et al., 2019	United Kingdom	Multicentre diagnostic accuracy	Emergency Department and Acute Medical Wards	785	81.4	Low
Vardy et al., 2020	United Kingdom	Quality improvement/implementation	Emergency Department	NR	NR	Moderate

All studies investigated the use of the 4AT in older adults, with inclusion criteria generally restricted to patients aged 65 years and over presenting to emergency care settings. It is worth noting that Pouw et al. collected data on the use of 4AT in both emergency care and on geriatric wards, and presented the data separately, so that analysis was not skewed by usage of the 4AT in ward settings. Four studies were conducted exclusively within EDs, while two included additional acute medical or geriatric ward settings [8-13].

Methodological approaches were heterogeneous. Three studies employed cohort or diagnostic accuracy designs, one was a large multicentre diagnostic accuracy study, and two were quality improvement or implementation studies [8-13]. Mean participant age ranged from 75 to 82 years where reported, reflecting the intended target population for delirium screening. Delirium prevalence varied considerably across studies, ranging from 6.1% to 60%, although not all studies reported this outcome.

Overall, the included studies represented a mixture of diagnostic accuracy, feasibility, and implementation research conducted across a range of emergency care contexts, providing evidence regarding both the practical application and performance of the 4AT in older adults.

Risk of Bias Results

The risk of bias across all six papers is outlined in Table 3. Three studies were judged to be at low risk of bias, two at moderate risk, and one at high risk of bias. Common methodological strengths included clearly defined eligibility criteria and appropriate outcome measurement. Frequently identified limitations included convenience sampling, incomplete reporting of implementation procedures, and limited consideration of potential confounding factors.

**Table 3:** Table showing judgement of bias based on Joanna Briggs Institute (JBI) criteria

Study	Key Strengths	Key Limitations	Overall Risk of Bias
Soler-Sanchis et al. (2024) [8]	Large sample, clear eligibility criteria, appropriate analysis	Limited reporting of missing data	Low
Pouw et al. (2023) [9]	Clear outcome measurement, appropriate reference standard	Small convenience sample	Moderate
Myrstad et al. (2022) [10]	Prospective design, clear administration procedures	Limited discussion of confounding factors	Low
Durant and Karia (2025) [11]	Clear implementation intervention	Small survey sample, no statistical analysis	High
MacLullich et al. (2019) [12]	Large multicentre design, robust methodology	Minor concerns regarding assessment timing	Low
Vardy et al. (2020) [13]	Real-world implementation study	No control group, limited participant detail	Moderate

Primary Outcomes

Table 4 summarises the primary feasibility outcomes reported across the six included studies. Overall, the evidence suggests that the 4AT is a feasible delirium screening tool for use in older adults presenting to EDs, demonstrating short administration times, minimal training requirements, generally positive staff perceptions, and successful integration into routine clinical workflows.

**Table 4:** Table showing primary feasibility outcomes of the included papers

Outcome	Synthesis of Findings	Supporting Studies
Completion time	The 4AT was consistently reported as a rapid screening tool. Where measured, administration took between approximately 1-3 minutes, with one study reporting a median completion time of 109 seconds and another reporting a mean time of 2.5 minutes. Integration into triage workflows did not appear to increase overall assessment time.	Soler-Sanchis et al. (2024) [8]; Pouw et al. (2023) [9]; Myrstad et al. (2022) [10]
Completion rate	Completion rates varied substantially between settings, ranging from 7% to 100%. Highest rates were observed in diagnostic accuracy studies where completion formed part of the study protocol, whereas implementation studies reported lower rates. Educational interventions and workflow integration improved completion rates over time.	Soler-Sanchis et al. (2024) [8]; Pouw et al. (2023) [9]; Myrstad et al. (2022) [10]; Durant and Karia (2025) [11]; MacLullich et al. (2019) [12]; Vardy et al. (2020) [13]
Staff acceptability	Staff generally viewed the 4AT positively, describing it as easy to use, practical, and suitable for busy emergency settings. Positive perceptions were reported among nurses, physicians, and non-specialist healthcare professionals. Some uncertainty remained regarding responsibility for screening and subsequent management following a positive result.	Pouw et al. (2023) [9]; Myrstad et al. (2022) [10]; MacLullich et al. (2019) [12]; Vardy et al. (2020) [13]; Durant and Karia (2025) [11]
Training requirements	Evidence consistently suggested that extensive training was not required for effective use of the 4AT. Several studies reported successful administration by clinicians with little formal delirium training, although brief introductory sessions were used in some settings to support implementation. Where training was implemented, it generally consisted of brief educational sessions or introductory lectures.	Pouw et al. (2023) [9]; Myrstad et al. (2022) [10]; MacLullich et al. (2019) [12]; Vardy et al. (2020) [13]; Durant and Karia (2025) [11]
Workflow impact	The 4AT appeared feasible to integrate into routine emergency care workflows. Studies reported no substantial increase in assessment burden and demonstrated improved delirium screening rates when supported by digital pathways, prompts, or structured implementation strategies. One study reported reduced emergency department length of stay among screened patients.	Soler-Sanchis et al. (2024) [8]; MacLullich et al. (2019) [12]; Vardy et al. (2020) [13]; Durant and Karia (2025) [11]
Barriers to implementation	Common barriers included time pressures, workload demands, communication difficulties, uncertainty regarding responsibility for screening, limited delirium knowledge, and lack of clarity regarding actions following a positive screen.	Soler-Sanchis et al. (2024) [8]; Durant and Karia (2025) [11]; MacLullich et al. (2019) [12]; Vardy et al. (2020) [13]
Facilitators to implementation	Facilitators included nurse-led screening, brief staff education, integration into electronic health records, leadership support, use of delirium champions, workflow prompts, and incorporation into existing triage processes.	Soler-Sanchis et al. (2024) [8]; Myrstad et al. (2022) [10]; Vardy et al. (2020) [13]; Durant and Karia (2025) [11]
Overall feasibility	Across all included studies, the 4AT was considered feasible for use in older adults attending emergency departments. Feasibility was supported by short administration times, minimal training requirements, positive staff perceptions, and successful integration into routine clinical practice when supported by organisational implementation strategies.	All included studies [8-13]

Administration time was reported in three studies and was consistently brief. Pouw et al. reported a median completion time of 109 seconds, while Myrstad et al. reported a median administration time of two minutes and a mean time of 2.5 minutes [9,10]. Soler-Sanchis et al. found that incorporation of the 4AT into nursing triage did not significantly increase assessment time compared with conventional triage procedures [8]. Collectively, these findings suggest that the 4AT can be completed rapidly within the time constraints of emergency care and is unlikely to impose substantial additional workload on clinical staff.

Completion rates varied considerably between studies. Diagnostic accuracy studies generally reported high completion rates, ranging from 58.4% to 100%, with MacLullich et al. achieving a completion rate of 99.2% across multiple acute care settings [8-10,12]. In contrast, implementation-focused studies reported lower completion rates, reflecting the challenges of embedding delirium screening into routine clinical practice. Durant and Karia reported a completion rate of 7% during their quality improvement project, while Vardy et al. observed completion rates increasing from 3% at baseline to 43% following implementation of a digital delirium pathway and a staff education programme [11,13]. These findings suggest that successful implementation may depend on organisational support and integration strategies rather than the usability of the tool itself.

Staff acceptability was generally positive across the included studies. Healthcare professionals described the 4AT as practical, straightforward, and suitable for use in busy emergency care environments [9,12]. Positive perceptions were reported among nurses, physicians, and other non-specialist healthcare professionals (i.e., staff without formal geriatric medicine or psychiatric training), supporting the use of the tool across different professional groups. However, implementation studies identified uncertainty regarding responsibility for conducting the assessment and managing positive screening results, highlighting potential organisational barriers to wider adoption [11-13].

No study reported a requirement for extensive or specialist training to use the 4AT, consistent with its design as a rapid, non-specialist screening tool. However, this does not mean training played no role in implementation: several studies incorporated brief education as part of successful rollout. Pouw et al. reported that the 4AT could be administered effectively by staff without prior delirium-specific training [9], while Myrstad et al. achieved good uptake following a brief 45-minute introductory session [10]. In the implementation studies, structured education and familiarisation sessions were used deliberately to support adoption [11-13]. Taken together, these findings suggest that while the 4AT itself demands little training to administer correctly, brief staff education is a recurring facilitator of successful implementation, rather than evidence that no training is beneficial.

Several barriers and facilitators to implementation were identified. Common barriers included time pressures, competing clinical priorities, communication difficulties, uncertainty regarding staff responsibilities, and limited confidence in the management of patients following a positive screening result [8,11-13]. Facilitators included integration into existing workflows, nurse-led triage assessment, educational interventions, electronic health record prompts, leadership support, and the use of delirium champions [8,11,13]. These findings suggest that organisational and contextual factors may play a greater role in successful implementation than characteristics of the screening tool itself.

Taken together, the available evidence indicates that the 4AT is a feasible delirium screening instrument for adults aged 65 years and older attending EDs. The tool can be administered rapidly, requires limited training, and is generally well accepted by clinical staff. While implementation challenges remain, particularly regarding workflow integration and staff engagement, targeted organisational strategies appear capable of improving uptake and supporting routine use in emergency care settings.

Secondary Outcomes 

The secondary outcomes collected from the six studies are summarised in Table 5. Diagnostic accuracy data were reported in three of the six included studies. Sensitivity estimates ranged from 67% to 85.1%, while specificity ranged from 66.9% to 95% [8,9,12]. Soler-Sanchis et al. reported the highest sensitivity (85.1%), suggesting that the 4AT identified a high proportion of patients with delirium when compared with DSM-V diagnostic criteria [8]. In contrast, MacLullich et al. reported the highest specificity (95%) in a large multicentre diagnostic accuracy study, indicating strong performance in correctly identifying patients without delirium [12].

**Table 5:** Table showing secondary outcomes of the included papers

Outcome	Synthesised Finding	Supporting Studies
Sensitivity	Ranged from 67% to 85.1%, indicating acceptable ability to identify delirium.	Soler-Sanchis et al. (2024) [8]; Pouw et al. (2023) [9]; MacLullich et al., (2019) [12]
Specificity	Ranged from 66.9% to 95%, suggesting good ability to exclude delirium when absent.	Soler-Sanchis et al. (2024) [8]; Pouw et al. (2023) [9]; MacLullich et al., (2019) [12]

Pouw et al. reported sensitivity and specificity values of 67% and 83%, respectively, in their evaluation of the Dutch version of the 4AT [9]. Although these estimates were lower than those reported elsewhere, the authors concluded that the tool remained clinically useful because of its brevity and ease of administration. Myrstad et al. primarily investigated feasibility and did not report sensitivity or specificity outcomes [10]. Similarly, implementation-focused studies conducted by Durant and Karia and by Vardy et al. did not evaluate diagnostic accuracy, instead focusing on uptake and integration into routine practice [11,13].

Overall, the available evidence suggests that the 4AT demonstrates acceptable diagnostic performance when compared with recognised reference standards, including Diagnostic and Statistical Manual of Mental Disorders IV (DSM-IV) and Diagnostic and Statistical Manual of Mental Disorders V (DSM-V) diagnostic criteria. However, the limited number of studies reporting diagnostic accuracy outcomes and variation in study designs preclude definitive conclusions regarding comparative performance across emergency care settings.

Discussion

Summary of Evidence

This rapid systematic review evaluated the feasibility of the 4AT for delirium screening among adults aged 65 years and older presenting to EDs. Across six included studies, the findings suggest that the 4AT is a feasible screening instrument that can be incorporated into routine emergency care with minimal burden on staff and workflow. The evidence demonstrated consistently short administration times, generally positive staff perceptions, and limited training requirements. Furthermore, several studies reported successful implementation by non-specialist healthcare professionals, supporting the intended design of the 4AT as a rapid delirium screening tool suitable for routine clinical practice.

Administration times were consistently brief where reported, ranging from approximately 109 seconds to 2.5 minutes. This is particularly relevant within EDs, where clinicians operate under significant time constraints and competing priorities. As detailed in the Results section, completion rates varied considerably by study type and setting, reflecting differences in organisational support rather than the usability of the 4AT itself.

Staff acceptability was generally favourable, with clinicians describing the 4AT as practical, straightforward, and appropriate for busy clinical environments. The evidence also suggested that extensive or specialist training is not required to administer the 4AT correctly; however, brief structured education was consistently associated with improved uptake, indicating that while the tool itself demands little training, its successful implementation benefits from some staff preparation. Barriers to implementation were primarily organisational, including workload pressures, uncertainty regarding responsibility for screening, and limited knowledge of delirium. Facilitators included educational interventions, workflow integration, electronic health record prompts, leadership support, and nurse-led screening approaches.

Although diagnostic accuracy was not the primary focus of this review, the available evidence suggested that the 4AT demonstrates acceptable diagnostic performance, with reported sensitivity ranging from 67% to 85.1% and specificity ranging from 66.9% to 95% [8,9,12]. These findings provide additional support for the use of the 4AT as a practical screening instrument within emergency care pathways.

Comparison With Existing Literature

The findings of this review are broadly consistent with the wider literature surrounding delirium screening and implementation of the 4AT. As outlined above, the 4AT has been promoted as a rapid screening tool requiring minimal training and suitable for use by non-specialist healthcare professionals. The present review supports these characteristics, demonstrating that the tool can be administered quickly and effectively within emergency care settings.

Previous systematic reviews evaluating delirium screening instruments have consistently identified practical barriers to implementation, particularly in acute and emergency care environments. Time constraints, competing clinical priorities, and limited staff confidence have frequently been reported as obstacles to routine delirium screening. The barriers identified in the present review mirror these findings, suggesting that implementation challenges remain despite growing recognition of the importance of delirium detection. Importantly, however, the studies included in this review indicate that these barriers are not necessarily attributable to the design of the 4AT itself but rather to broader organisational and operational factors.

The finding that educational interventions and workflow integration improve uptake is also consistent with implementation science literature. Previous research has demonstrated that embedding screening tools within existing clinical pathways and electronic health record systems can improve compliance with recommended assessments. Similarly, the positive impact of nurse-led triage screening observed in some studies aligns with wider evidence supporting early identification of delirium during the initial stages of ED assessment.

The diagnostic accuracy findings identified in this review are broadly comparable to those reported in previous evaluations of the 4AT. While sensitivity and specificity estimates varied between studies, overall performance remained within acceptable ranges for a screening instrument intended to identify patients who require further assessment. This supports existing recommendations advocating the use of the 4AT as part of routine delirium detection strategies in older adults.

Strengths and Limitations

Several limitations should be considered when interpreting the findings of this review. First, this review searched three major databases (PubMed, MEDLINE, EMBASE); other sources (e.g., CINAHL, Web of Science, trial registries) were not searched given the rapid review timeframe, which may have resulted in missed studies.

Second, the evidence base was relatively small, with only six studies meeting the inclusion criteria. This limited the ability to draw definitive conclusions and reduced opportunities for subgroup analysis.

Third, substantial heterogeneity existed between studies in terms of design, sample size, setting, implementation approach, and outcome reporting. While all studies evaluated the 4AT in emergency care contexts, differences in methodology and reporting limited direct comparison between findings. As a result, quantitative synthesis was not appropriate and a narrative approach was adopted.

Fourth, reporting of sample size was incomplete or difficult to attribute to the ED setting specifically in two of the six included studies. Vardy et al. did not report a sample size, which limits interpretation of their reported completion-rate increase (from 3% to 43%) since the denominator underlying these percentages is unknown [13]. Pouw et al. recruited across both ED and geriatric ward settings without reporting an ED-specific participant count, meaning the total combined sample size across included studies (1,583) is an approximation rather than a precise count of ED-only participants [9]. These gaps constrain confidence in the overall scale of evidence specifically applicable to ED practice.

Fifth, many feasibility outcomes were inconsistently reported. Completion time, staff acceptability, workflow impact, and training requirements were not measured using standardised approaches across studies. In several cases, outcomes were reported qualitatively rather than quantitatively, reducing the ability to compare findings across studies.

Sixth, the methodological quality of included studies varied. Although several studies were assessed as having a low risk of bias, others were judged to have moderate or high risk due to issues such as convenience sampling, limited participant numbers, incomplete reporting, and lack of statistical analysis. Consequently, some findings should be interpreted with caution.

In addition, reference standards used to assess diagnostic accuracy varied across studies, with DSM-IV and DSM-V criteria applied in different validation studies. These diagnostic criteria may not directly reflect delirium diagnostic practice within UK EDs, which limits the generalisability of the reported sensitivity and specificity figures to routine UK ED clinical practice.

Finally, the review focused specifically on studies involving older adults in emergency care settings. While this improves relevance to the review question, the findings may not be generalisable to other clinical settings such as inpatient wards, community services, or long-term care facilities.

Implications

The findings of this review have several implications for clinical practice. Delirium remains under-recognised in EDs despite its association with increased mortality, prolonged hospitalisation, functional decline, and institutionalisation. Early identification is therefore essential to enable timely investigation and management of underlying causes.

The evidence suggests that the 4AT is well suited to ED environments because it can be completed rapidly and requires limited training. This may be particularly valuable in departments experiencing increasing demand, workforce pressures, and time constraints. The ability to administer the assessment without specialist expertise also supports wider implementation across multidisciplinary teams, including nurses, physicians, and other healthcare professionals involved in the care of older adults.

Importantly, the review suggests that successful implementation is likely to depend on organisational support rather than solely on selection of the screening tool. Educational initiatives, electronic prompts, integration into routine documentation, and clearly defined staff responsibilities appear to be important facilitators of sustained uptake. Healthcare organisations seeking to improve delirium detection should therefore consider implementation strategies alongside adoption of the 4AT itself.

The findings may also have implications for compliance with national and international guidance that recommends routine delirium screening among older adults. By demonstrating feasibility within emergency care settings, the review provides evidence supporting the incorporation of the 4AT into standard assessment pathways for patients aged 65 years and older.

Further research is required to strengthen the evidence base regarding the feasibility and implementation of the 4AT in EDs. Future studies should employ standardised measures of feasibility outcomes, including completion time, completion rates, staff acceptability, and workflow impact. Consistent reporting would facilitate comparison between studies and improve opportunities for evidence synthesis.

Additional implementation research is also needed to identify the most effective strategies for embedding delirium screening into routine emergency care. Comparative evaluations of educational interventions, electronic health record integration, nurse-led screening models, and multidisciplinary implementation approaches would be particularly valuable.

## Conclusions

This rapid systematic review found that the 4AT is a feasible delirium screening tool for adults aged 65 years and older presenting to EDs. Across the six included studies, the 4AT demonstrated short administration times, minimal training requirements, and generally positive staff acceptability. While completion rates varied between settings, evidence suggests that successful implementation is influenced more by organisational factors, such as staff education, workflow integration, and electronic prompts, than by limitations of the tool itself.

Diagnostic accuracy findings provide some additional support for the 4AT as a screening instrument, although the available evidence addresses implementation characteristics more extensively than clinical outcome data, reflecting a gap that future research should address. Given the methodological limitations present across several of the included studies - including convenience sampling, incomplete reporting, and the small number of studies overall - these findings should be regarded as preliminary rather than conclusive. Future research should focus on standardised reporting of feasibility outcomes and evaluation of implementation strategies to support consistent uptake. Overall, the available evidence is encouraging and supports further consideration of the 4AT as a practical tool for improving delirium detection among older adults in emergency care settings, pending stronger and more consistent evidence.

## Appendices

Appendix 1 - PICO framework

This table demonstrates how the PICO model helped us develop the research question to be answered by this systematic review.

**Table 6:** PICO framework used to develop research question in this systematic review

PICO Criterion	Details
Population	Adults aged ≥65 presenting to any Emergency Department
Intervention/index test	The 4AT assessment tool [5]
Comparator	No formal comparator (descriptive feasibility focus); diagnostic accuracy data extracted as secondary outcome where reported
Outcomes – primary	Completion time (mean/median minutes); completion rate (%); staff acceptability (Likert or qualitative); training requirements; barriers and facilitators to implementation
Outcomes - secondary	Sensitivity and specificity vs reference standard

Appendix 2 - Search strategies

Searches were conducted across three electronic databases in April 2026. The following strategies are presented in full to ensure reproducibility of the review.

Appendix 1a - PubMed

Database: PubMed Interface: National Library of Medicine (www.pubmed.ncbi.nlm.nih.gov) Date searched: 21/04/2026 Records retrieved: 25

Search string:

("4AT" OR "four AT" OR "4 AT test") AND ("emergency department" OR "emergency room" OR "accident and emergency" OR "A&E") AND ("older adult" OR "elderly" OR "geriatric" OR "aged") AND ("2011"[PDAT] : "3000"[PDAT]) AND (English[lang]) NOT ("review"[PT] OR "editorial"[PT] OR "letter"[PT] OR "comment"[PT])

Language: English only

Date: January 2011 to present

Publication types excluded: reviews, editorials, letters, comments

Appendix 1b - MEDLINE (Ovid)

Database: Ovid MEDLINE(R) ALL Interface: Ovid SP Date searched: 21/04/2026 Records retrieved: 30

**Table 7:** Search strategy used for MEDLINE (Ovid)

Line	Search Term	Field
1	"4AT"	.mp.
2	"four AT"	.mp.
3	"4 AT test"	.mp.
4	1 OR 2 OR 3	4AT concept set
5	exp Emergency Service, Hospital/	MeSH exploded
6	"emergency department*"	.mp.
7	"emergency room*"	.mp.
8	"accident and emergency"	.mp.
9	"A&E"	.ti,ab.
10	5 OR 6 OR 7 OR 8 OR 9	ED concept set
11	exp Aged/	MeSH exploded
12	exp Aged, 80 and over/	MeSH exploded
13	"older adult*"	.mp.
14	"elder*"	.mp.
15	"geriatric*"	.mp.
16	11 OR 12 OR 13 OR 14 OR 15	Age concept set
17	4 AND 10 AND 16	Combined set
18	limit 17 to (English language and yr="2011-Current")	Language and date limits
19	limit 18 to (editorial or letter or review or comment)	Exclusion types identified
20	18 NOT 19	Final search: 30

Language: English only

Date: January 2011 to present

Publication types excluded via line 19: editorials, letters, reviews, comments

Appendix 1c - EMBASE (Ovid)

Database: Embase Interface: Ovid SP Date searched: 21/04/2026 Records retrieved: 140

**Table 8:** Search strategy used for EMBASE (Ovid)

Line	Search Term	Field
1	"4AT"	.mp.
2	"four AT"	.mp.
3	"4 AT test"	.mp.
4	1 OR 2 OR 3	4AT concept set
5	exp emergency health service/	Emtree exploded
6	exp emergency ward/	Emtree exploded
7	"emergency department*"	.mp.
8	"emergency room*"	.mp.
9	"accident and emergency"	.mp.
10	"A&E"	.ti,ab.
11	5 OR 6 OR 7 OR 8 OR 9 OR 10	ED concept set
12	exp aged/	Emtree exploded
13	exp very elderly/	Emtree exploded
14	"older adult*"	.mp.
15	"elder*"	.mp.
16	"geriatric*"	.mp.
17	12 OR 13 OR 14 OR 15 OR 16	Age concept set
18	4 AND 11 AND 17	Combined set
19	limit 18 to (english language and yr="2011-Current")	Language and date limits
20	limit 19 to (editorial or letter or review or erratum)	Exclusion types identified
21	19 NOT 20	Final search: 140

Language: English only

Date: January 2011 to present

Publication types excluded via line 20: editorials, letters, reviews, errata

Appendix 3 - Full-text exclusion list

A total of 17 records were excluded following full-text assessment. Reasons for exclusion are documented below. Where a study met more than one exclusion criterion, all applicable reasons are noted.

**Table 9:** Table showing reasons for full-text exclusion

No.	Author(s)	Year	Title	Reason(s) for Exclusion
1	Gagné et al.	2018	Performance of the French version of the 4AT for screening the elderly for delirium in the emergency department	No feasibility data - diagnostic accuracy outcomes only
2	Niu et al.	2024	Wake-up Call - High Delirium Prevalence in Older Adults Boarded in Emergency Department	Conference abstract; no feasibility data - prevalence outcomes only
3	Manners et al.	2015	Improving Delirium Care in the Medical Assessment Unit	Conference abstract - no full-text article available
4	Ahmed and Honney	2024	Implementation of 4AT and Delirium Bundle in Patient Management - A Quality Improvement Project	Conference abstract; no feasibility data - outcomes limited to bundle utilisation only
5	Williams et al.	2024	Audit of Geriatric Emergency Medicine-Unit Staff Compliance in Providing Care in Line with the Age Friendly Health Systems Conceptual Framework	Conference abstract; not 4AT-specific
6	Niranjan and Findlay	2024	Implementing a Comprehensive Geriatric Assessment (CGA) in Older Adults Presenting to a District General Emergency Department	Conference abstract; no feasibility data - wrong outcome reported
7	Meldon and Saxena	2024	Delirium Screening: Prevalence and Outcomes in Older Emergency Department Patients Across a Large Healthcare System	Conference abstract - no full-text article available
8	Sheil and Okon	2023	Prevalence of Delirium Among Older Patients Attending a Frailty Service in an Emergency Department	Conference abstract; no feasibility data - prevalence outcomes only
9	Wong and Hor	2022	Assessing for Cognitive Impairment in Older People: A Quality Improvement Project	Conference abstract - no full-text article available
10	Spyraki and Karpetas	2021	Cognitive Assessment Using 4AT	Conference abstract; no feasibility data - not a primary study
11	Patton and O'Sullivan	2021	Optimizing the 'G' in Emergency Medicine - Delirium Screening by ED Physicians	Conference abstract - no full-text article available
12	Tan and Williams	2021	Are Older Patients in Ireland Being Adequately Screened for Delirium? Results from an Irish Peripheral Hospital	Conference abstract - setting not exclusively ED
13	Saxena and Ancheta	2020	Detection of Delirium Among Older Adults in the Emergency Department Through the Utilisation of 4AT	Conference abstract - no full-text article available
14	Torsney and Romero-Ortuno	2019	A Service Evaluation of the Performance of the 4AT as a Cognitive Screening Tool in an English University Hospital	Conference abstract - no full-text article available
15	Myrstad and Alaburic	2018	Rapid Cognitive Assessment with 4AT in Acutely Ill Patients Aged ≥65 Years with Suspected Infection	Conference abstract; no feasibility data - wrong outcome reported
16	O'Reilly and Moloney	2017	Feasibility of the 4AT Rapid Assessment Tool for Delirium Screening in the Older Patient in the Emergency Department	Conference abstract - no full-text article available
17	Vale and Bird	2019	Pop-up Delirium Simulation Training	Conference abstract - no full-text article available
